# Quality of Life and Toxicity after SBRT for Organ-Confined Prostate Cancer, a 7-Year Study

**DOI:** 10.3389/fonc.2014.00301

**Published:** 2014-10-28

**Authors:** Alan Jay Katz, Josephine Kang

**Affiliations:** ^1^Flushing Radiation Oncology Services, Flushing, NY, USA; ^2^Department of Medicine, NYU Langone Medical Center, New York, NY, USA

**Keywords:** prostate cancer, stereotactic body radiation therapy, quality of life, SBRT

## Abstract

**Objectives:** Stereotactic body radiation therapy (SBRT) yields excellent disease control for low- and intermediate-risk prostate cancer by delivering high doses of radiation in a small number of fractions. Our report presents a 7-year update on treatment toxicity and quality of life (QOL) from 515 patients treated with prostate SBRT.

**Methods:** From 2006 to 2009, 515 patients with clinically localized, low-, intermediate-, and high-risk prostate cancer were treated with SBRT using Cyberknife technology. Treatment consisted of 35–36.25 Gy in 5 fractions. Seventy-two patients received hormone therapy. Toxicity was assessed at each follow-up visit using the expanded prostate cancer index composite (EPIC) questionnaire and the radiation therapy oncology group urinary and rectal toxicity scale.

**Results:** Median follow-up was 72 months. The actuarial 7-year freedom from biochemical failure was 95.8, 89.3, and 68.5% for low-, intermediate-, and high-risk groups, respectively (*p* < 0.001). No patients experienced acute Grade 3 or 4 acute complications. Fewer than 5% of patients had any acute Grade 2 urinary or rectal toxicity. Late toxicity was low, with Grade 2 rectal and urinary toxicity of 4 and 9.1%, respectively, and Grade 3 urinary toxicity of 1.7%. Mean EPIC urinary and bowel QOL declined at 1 month post-treatment, returned to baseline by 2 years and remained stable thereafter. EPIC sexual QOL declined by 23% at 6–12 months and remained stable afterwards. Of patients potent at baseline evaluation, 67% remained potent at last follow-up.

**Conclusion:** This study suggests that SBRT, when administered to doses of 35–36.25 Gy, is efficacious and safe. With long-term follow-up in our large patient cohort, we continue to find low rates of late toxicity and excellent rates of biochemical control.

## Introduction

Several recent studies on stereotactic body radiotherapy (SBRT) for prostate cancer have been published, providing evidence of excellent biochemical disease control with short-term follow-up (1–6). With follow-up of up to 5 years, reported bowel and bladder toxicity has been quite low, and quality of life (QOL) measures have been favorable (7). However, the likelihood of long-term toxicity is not well known, and more studies with longer follow-up are necessary in order to confirm initial reports. As a result, the adoption of prostate SBRT in the clinic has proceeded slowly, though it has recently been accepted by both the American society for radiation oncology (ASTRO) and the national comprehensive cancer network (NCCN). The purpose of our study is to document incidence of bowel, bladder, and sexual toxicity and QOL in a large cohort of patients, with follow-up as long as 8 years. This represents significantly longer follow-up than previously published.

## Materials and Methods

### Patient selection

Between 2006 and 2009, 515 consecutive patients with newly diagnosed, non-metastatic, biopsy-proven prostate cancer were treated with SBRT. The first 15 patients were treated on an IRB-approved in-house protocol, and the remainder was treated off protocol, with similar treatment parameters. All patients were consented for treatment and agreed to use of follow-up data for research purposes. Patients were stratified into D’Amico risk groups (low-risk: PSA < 10 and Gleason sum of 6 and clinical stage T1c–T2a, intermediate-risk: PSA 10–20 or Gleason sum of 7 or clinical stage T2b, high risk: PSA > 20 or Gleason sum of 8–10 or clinical stage T2c or higher). Seventy-two patients received androgen deprivation therapy (ADT) prior to and during treatment, at the discretion of the urologist.

### Treatment

Fiducial-based image-guided SBRT was delivered using the CyberKnife system (Accuray Inc., Sunnyvale, CA, USA), G3 model with multiplan 2.0. The treatment specifics of Cyberknife have been published previously (8). General techniques are outlined here. Four gold fiducials were placed transperineally with ultrasound guidance into the prostate. This was followed by a non-contrast CT scan in the supine position, with alpha cradle immobilization. CT scan was obtained using a 64 slice CT scanner with 1.25 mm slices. Unless contraindicated, patients underwent prostate MRI, and images were fused to CT images in order to better visualize the inferior portion of the prostate. No urinary catheter was used. Dose was prescribed to the planning target volume (PTV), which consisted of a 5 mm expansion on the prostate, reduced to 3 mm posteriorly. Homogeneous planning was performed, and dose normalized to the 83–87% isodose line, with full prescription dose covering at least 95% of the PTV. Anatomical contours of the prostate, seminal vesicles, rectum, bladder, penile bulb, femoral heads, and testes were generated and dose volume histograms (DVH) constructed. The urethra was not contoured as no constraints were placed on it. Rectal DVH goals were V50 < 50% (i.e., the volume receiving 50% of the prescribed dose was <50%), V80 < 20, V90 < 10, and V100 < 5%. The bladder DVH goals were V50 < 40 and V100 < 10%. A typical D50 for the bladder and rectum was 40–45% of the maximum dose. The femoral head DVH goal was V40 < 5%.

During a typical 45-min treatment, fiducial seeds were tracked and positional adjustments made at 30–60 s intervals. Every morning prior to SBRT, patients underwent bowel prep with Dulcolax^®^ (Boehringer Ingelheim, Germany) and a Fleet^®^ Enema (C.B. Fleet Company, Inc., Lynchburg, VA, USA). In addition, all patients received 1500 mg of amifostine (MedImmune, LLC, Gaithersburg, MD, USA), mixed in saline and instilled into the rectum approximately 15–20 before treatment (9). The radiation dose was 35 (*n* = 158) or 36.25 (*n* = 357) Gy in 5 fractions, given daily. The first 50 patients treated received 35 Gy. After a report from Stanford documenting the feasibility of using 36.25 Gy (10), we increased the dose accordingly for the next 30 months. Dose was reduced back to 35 Gy due to increased toxicity.

### Follow-up

The median follow-up for the entire cohort was 72 months. PSAs were obtained at baseline. Post-treatment, PSA was obtained at 3 months post-treatment intervals for the first 2 years, and at 6 months intervals thereafter. The Phoenix definition (nadir + 2) was used to define relapse (11).

Urinary, sexual, and bowel QOL expanded prostate cancer index composite (EPIC) scores (12) were obtained from patients at baseline, 3 weeks post-treatment, and subsequently every 3–6 months for the first 2 years, then 12 months. Acute and late bowel and bladder toxicity was scored according to the criteria set forth by radiation therapy oncology group (RTOG) (13).

### Statistical analyses

Actuarial biochemical control was calculated using the Kaplan–Meier method and log-rank analysis performed. The likelihood ratio test was used to determine differences in toxicity. Cox multivariate regression analysis was used to analyze the patient factors associated with development of Grade 2–3 late urinary toxicity.

## Results

### Patient characteristics

Patient characteristics are summarized in Table 1. The median follow-up for all patients was 72 months (range, 0–96 months), 26 patients had follow-up for as long as 96 months.

**Table 1:** **Patient characteristics**.

Age at diagnosis	Years	
Mean	68.5 (43.8–89.2)	
Median	69.0 (43.8–89.2)	
Age at diagnosis	Number of patients	Percent of patients
40–49	4	0.7
50–59	77	15.0
60–69	201	39.0
70–79	196	38.1
80–89	37	7.2
PSA level at treatment	ng/mL	
Combined mean (range)	6.6 (1.0–42.9)	
Median	5.4	
PSA level at diagnosis	Number of patients	Percent of patients
<4 ng/mL	83	16.1
4–10 ng/mL	368	71.5
>10–20 ng/mL	64	12.4
Risk Category		
Low	324	62.9
Intermediate	153	29.7
High	38	7.4
Clinical Stage		
T1a	2	0.4
T1c	462	89.7
T2a	51	9.9
Gleason Score		
6	357	69.3
7 (3 + 4)	84	16.3
7 (4 + 3)	42	8.2
8 (4 + 4)	24	4.7
9 (4 + 5)	6	1.1
9 (5 + 4)	2	0.4
Hormone Treatment		
No	443	86.0
Yes	72	14.0
RT Treatment		
35 Gy	158	30.7
36.25 Gy	357	69.3

Using D’Amico risk stratification, 324 patients were low-risk, 153 were intermediate-risk, and 38 were high-risk. Median patient age was 69.5 (range, 43.8–89.3 years). The median PSA at diagnosis was 5.4 ng/mL. At last follow-up, 59 patients were deceased, none from prostate cancer. There were 13 deaths in the 35 Gy group and 46 deaths in the 36.25 Gy group, none attributable to prostate cancer. Seventy-two patients received ADT. Of this group, 26 patients were low-risk, 25 were intermediate-risk, and 21 were high-risk.

### Acute toxicity

All patients were seen at 3 weeks and 3 months post-treatment. At each visit, patients were assessed for bowel and bladder toxicity using RTOG criteria. Overall, 4% of patients reported Grade 2 urinary or bowel toxicity. Of patients who received 35 Gy, Grade 1 and Grade 2 urinary toxicity was 72 and 4%, respectively, and Grade 1 and Grade 2 rectal toxicity was 76 and 4%, respectively. There was no Grade 3–4 toxicity observed.

Of patients who received 36.25 Gy, Grade 1 and Grade 2 urinary toxicity was 74 and 4%, respectively, and Grade 1 and Grade 2 rectal toxicity was 78 and 4%, respectively. There was no significant difference in the incidence of early toxicity for the two doses used.

### Late toxicity – sexual potency

Patients were assessed for sexual potency prior to start of treatment, 375 out of 515 patients reported themselves to be potent. Of this group, 252 (67.2%) remained potent at last follow-up, with 25% requiring medication. Of the 375 patients initially potent at baseline, 37 received ADT, and only 14 out of 37 (38%) remained potent at last follow-up. In contrast, 338 patients who were potent at baseline did not receive ADT, and 239 out of 338 (70.7%) remained potent at last follow-up. The difference was statistically significant, *p* < 0.001.

### Late toxicity – urinary

**Figure 1:**
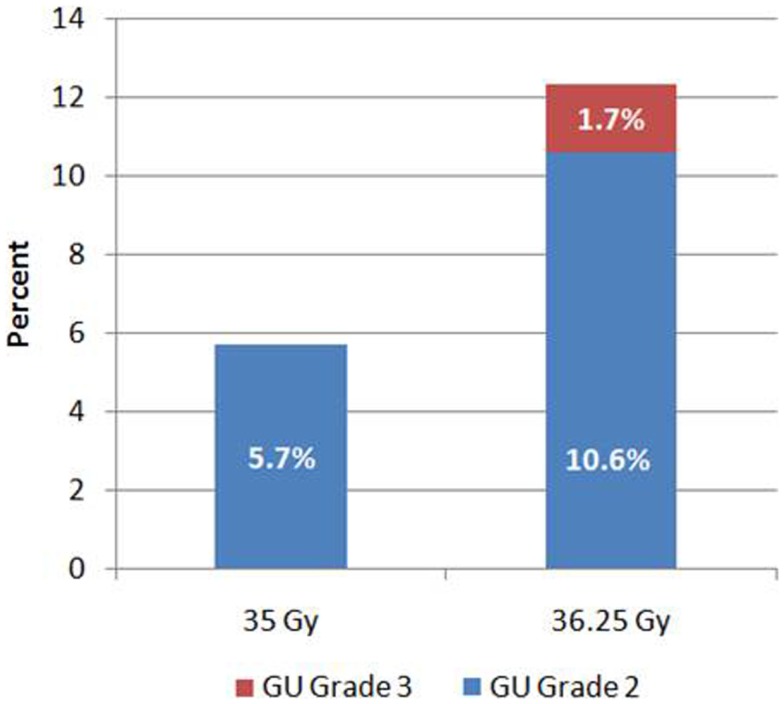
**Genitourinary toxicity by dose**.

Grade 2 urinary toxicity (mostly severe dysuria, urgency, or obstructed flow) occurred in 47 (9.1%) patients. Of the 158 patients who received 35 Gy, 9 (5.7%) had Grade 2 urinary toxicity. In comparison, of the 357 patients who received 36.25 Gy, 38 (10.6%) developed Grade 2 urinary toxicity (Figure 1). Grade 3 toxicity was significantly higher after 36.25 Gy compared to 35 Gy (*p* = 0.005), as was the overall incidence of Grade 2 or higher toxicity (*p* = 0.05). Late Grade 3 toxicity occurred in nine patients (1.7%) and consisted of either urinary retention requiring surgery or bleeding requiring laser coagulation. All Grade 3 events occurred in patients who received 36.25 Gy.

The median time to development of late urinary toxicity was 18 months (range, 6–60 months). Only 5 of 47 patients (11%) developed urinary toxicity after 36 months. At last follow-up, 37 of the 47 patients had complete resolution of their symptoms.

### Late toxicity – gastrointestional

**Figure 2:**
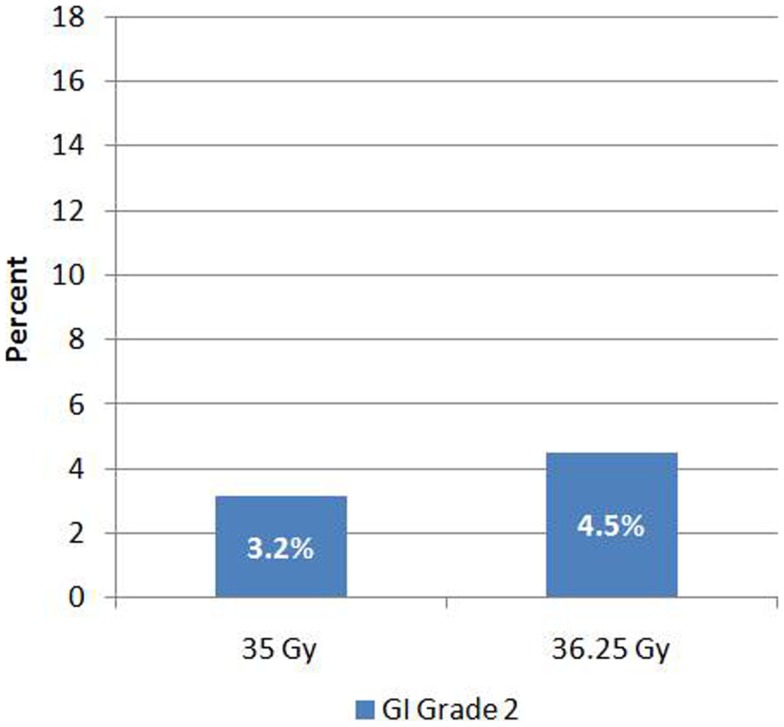
**Gastrointestinal toxicity by dose**.

Late Grade 2 bowel toxicity occurred in 21 (4%) of patients. Of this group, five patients were on anticoagulation therapy with Coumadin. Most common manifestation was rectal bleeding (85.7%). Grade 2 toxicity occurred in 3.2% (*n* = 5) and 4.5% (*n* = 16) of patients who received 35 and 36.25 Gy, respectively (Figure 2). This difference was not significant (*p* = 0.48). There was no Grade 3–4 bowel toxicity noted. The median time to development of bowel toxicity was 18 months (range, 6–44 months). At last follow-up (median follow-up = 72 months), 19 of the 21 affected patients experienced resolution of their symptoms.

### Prostate volume

Data on prostate volume were available for 336 patients. Median prostate volume was 59.15 cc (16.8–224 cc), and mean prostate volume was 65.3 cc (SD = 30.2 cc). Patients were analyzed based on prostate volume greater than or less than 60 cc, 173 patients had prostate volume ≤60 cc versus 163 with prostate volume >60 cc. There was higher incidence of both Grade 2 (*n* = 19, 11.6%) and Grade 3 (*n* = 5, 3.1%) toxicity in patients with prostate volume >60 cc, compared to Grade 2 (*n* = 12, 6.9%) and Grade 3 (*n* = 1, 0.6%) toxicity in patients with prostate volume ≤60 cc, this trended toward significance (*p* = 0.051). There was no significant difference in GI toxicity between patients with prostate volume >60 cc (Grade 2 toxicity *n* = 7, 4.3%) versus those with prostate volume ≤60 cc (Grade 2 toxicity *n* = 6, 3.7%).

### Quality of life – EPIC questionnaire

**Figure 3:**
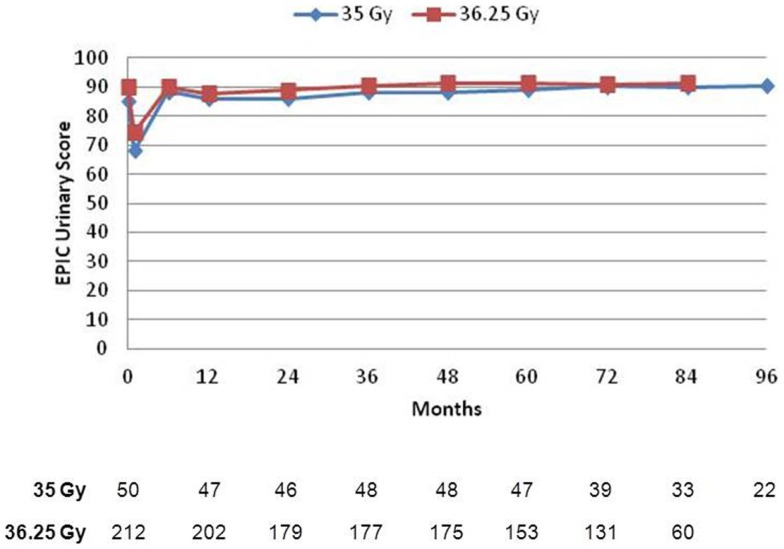
**Expanded prostate cancer index composite urinary scores**. The mean EPIC score is depicted for each time point. A number of patients with completed questionnaires are shown in table below.

**Figure 4:**
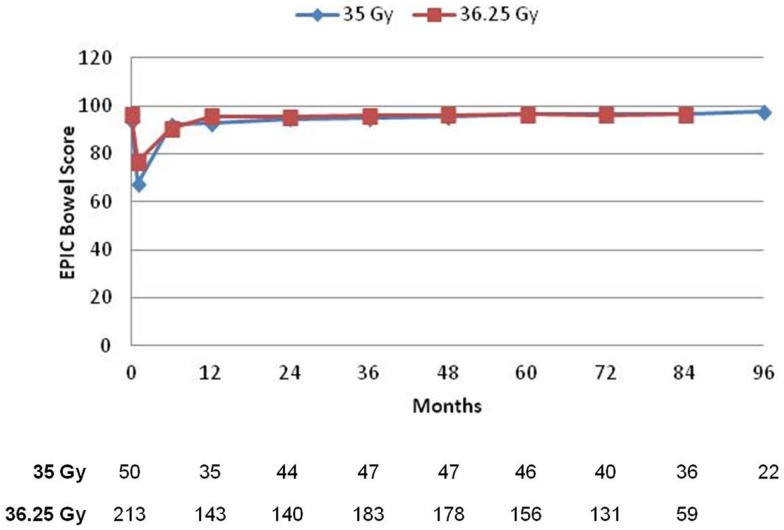
**Expanded prostate cancer index composite bowel scores**. The mean score is depicted for each time point. A number of patients with completed questionnaires are shown in table below.

**Figure 5:**
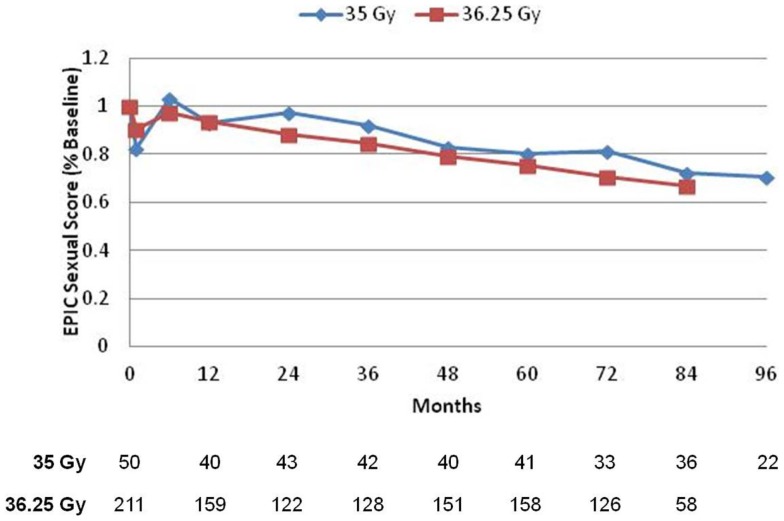
**Expanded prostate cancer index composite sexual scores**. EPIC sexual scores are reported as a percentage of each respective patient’s baseline score. A number of patients with completed questionnaires are shown in table below.

Prior to treatment, all patients completed the initial EPIC questionnaire to evaluate urinary, bowel, and sexual QOL. At each subsequent time points, patients were requested to fill out the EPIC questionnaire to assess follow-up QOL, but not all patients were compliant. Mean scores for all three domains, with the number of patients responding at each follow-up interval, is depicted in Figures 3–5. For urinary (Figure 3) and bowel (Figure 4) domains, mean EPIC scores decreased acutely and then gradually rose back to baseline by one year. After 1 year, mean EPIC scores extending out to 8 years did not differ significantly from baseline. EPIC sexual QOL (Figure 5) declined by 23% at 6–12 months and remained stable, 67% of the patients potent at baseline remained potent at last follow-up.

There was no significant difference in EPIC bowel, sexual, or urinary scores between 35 or 36.25 Gy at any time point.

### Multivariate analysis

On univariate analysis, prostate size >60 cc was found to be a significant (*p* = 0.03) predictor of Grade 2 or higher late GU toxicity (Table 2). Using a higher dose trended toward significance (*p* = 0.051), whereas baseline GU EPIC score was not significant. Multivariate analysis was performed looking at these three variables. Dose of 36.25 versus 35 Gy was the only significant variable, with *p* < 0.0001 (RR 3.31, 95% CI 2.17–5.35).

**Table 2:** **Univariate (UVA) and multivariate (MVA) logistic regression analyses looking at patient characteristics and the effect on Grade 2 or higher late GU toxicity**.

Factor	UVA	MVA	
	*p*	*p*	RR (95% CI)
Prostate size (above or below 60 cc)	0.03	0.03	0.86 (0.66–1.13)
Dose (35 versus 36.25 Gy)	0.051	<0.0001	3.31 (2.17–5.35)
Baseline GU EPIC score (above or below 90)	0.39	0.58	0.93 (0.71–1.21)

### Biochemical control and PSA

**Figure 6:**
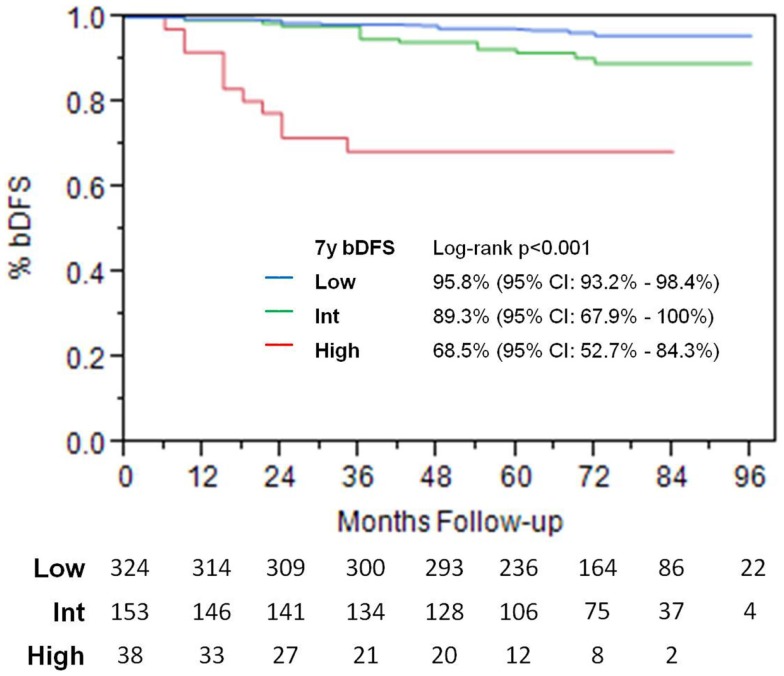
**Biochemical disease-free survival**.

Actuarial 7-year biochemical recurrence-free survival 95.6, 89.6, and 68.5% was 95.6% for low-risk, 89.6% for intermediate-risk, and 68.5% for high-risk patients (Figure 6).

For low- and intermediate-risk patients, there was no significant difference in biochemical disease-free survival (bDFS) between 35 versus 36.25 Gy (*p* = 0.36).

## Discussion

This study has the longest toxicity and QOL follow-up to date for prostate SBRT, and as a result, several important questions can begin to be addressed. The first question is whether SBRT doses of 35–36.25 Gy can be well-tolerated. Prior reports on SBRT, with only several years follow-up, reported excellent QOL outcomes but raised skepticism that late toxicity events would occur after many years. Our results suggest that the incidence of bowel and bladder toxicity continues to remain low, even after extended follow-up. Interestingly, over 90% of toxicity events occurred within 3 years of treatment, suggesting that the incidence of late toxicity will continue to remain low as more follow-up is obtained. Our low toxicity and excellent QOL are comparable or lower than results published for HDR and LDR brachytherapy, proton beam, and dose-escalated IMRT (14–17).

The low toxicity is consistent with radiobiologic data suggesting that prostate cancer has a very low α/β ratio, whereas the normal bowel/bladder tissue has α/β ratio of 3 (18), making hypofractionation a logical treatment choice. Assuming α/β ratio of 3 (18) for late soft tissue complications, the radiation doses used in this study of 35–36.25 Gy are equivalent to delivering 70–77 Gy at 1.8 Gy per fraction to surrounding normal tissue, this is lower than the dose to normal tissues delivered using standard IMRT. In addition, the ability to utilize tighter margins for SBRT treatment allows for more favorable bladder and rectum DVHs, and translates to mild bowel and bladder toxicity in both the short- and long-term.

In a recent well-publicized article by Yu et al (19), it has been suggested that SBRT may cause more urinary side effects than IMRT. Our data show a toxicity profile and QOL that compares favorably to IMRT. For instance, our overall rate of 9% Grade 2 urinary toxicity is comparable to Zelefsky’s report of 9% Grade 2 toxicity in 2006 (20). In fact, our rate of 5.7% late Grade 2 toxicity with 35 Gy, with no Grade 3 events, is arguably better than IMRT (21). Although it is not known, it is likely that patients treated to higher doses were included in the study by Yu. Based on our findings, which suggest 36.25 Gy results in greater toxicity than 35 Gy, we surmise that higher doses will result in even greater rates of toxicity, and likely account for Yu’s findings.

Our study also begins to address the question of appropriate SBRT dose. We noted a significant increase in urinary toxicity as the dose rises from 35 to 36.25 Gy. This is equivalent to normal tissue EQD increase from 70 to 77 Gy. Delivering 35 Gy in 5 fractions is equivalent to 200 Gy BED to the tumor cells, and studies suggest going higher than this dose does not result in better disease control (22). As a result, we hypothesize that 35 Gy may be the optimal dose for low- and intermediate-risk prostate cancer, and higher doses may result in increased toxicity without increased biochemical control. Recently, published studies on prostate SBRT, documenting unacceptably high rates of toxicity with higher radiation doses, are consistent with this hypothesis (23), and merits further study. Of note, the current RTOG 0938 trial is treating patients to dose of 36.25 in 5 fractions, and our data suggest that there may be greater toxicity with this higher dose.

Despite the higher toxicity noted in patients treated to 36.25 Gy, the QOL data did not reflect any disparity between the two groups by dose. Most bowel and bladder toxicity resolved with time and symptomatic treatment; as a result, QOL was not impacted for significant lengths of time and in most cases had returned to patient baseline once toxicity resolved. We believe this likely explains why QOL data between the two doses remained similar, whereas RTOG toxicity was significantly higher in the 36.25 Gy patient cohorts though the incidence of toxicity was greater with the higher dose.

The potential impact of prostate size and pre-treatment urinary function on long-term urinary toxicity has been an interesting question for study. To address this, a multivariate analysis was performed, and neither gland size nor impaired baseline urinary QOL predicted for late GU toxicity. These results are reassuring and suggest large prostate size and baseline poor urinary function should not automatically preclude patients from being considered for SBRT.

Regardless of dose, rectal toxicity remained quite mild. We do not believe the low rate of rectal toxicity can be attributed to the use of amifostine. Other groups have published reports on their prostate SBRT experience, and have also found low toxicity without the use of Amifostine (1, 3, 8). In fact, in our clinic, use of Amifostine was discontinued since early 2010, and thus far, we have not observed any increases in rectal toxicity.

Finally, we report encouraging potency preservation rates, with 67% of initially potent at baseline able to retain good function. This is similar to outcomes for HDR brachytherapy (16) and more favorable than outcomes reported for dose-escalated IMRT (15). In fact, potency preservation rates after SBRT are only slightly worse than what one would expect in a similar cohort of men in this age group, who did not receive any radiotherapy (24). The decrease in potency is reflected in the overall reduction of sexual QOL scores. Since no significant difference in sexual QOL was observed between the doses used, we postulate this is due to the tight constraints to the penile bulb that were utilized for both doses, and conjecture that penile bulb dose may be an important predictor of potency preservation.

It should be noted that we delivered all treatments daily (QD), Monday through Friday. King suggested in his 2009 article that every other day (QOD) treatments to 36.25 Gy yields less rectal toxicity, compared to QD (10). He reported 18% Grade 2 rectal toxicity in patients treated QD, significantly higher than our findings, which suggests that there may have been a dosimetric issue with these early patients. In a more recent 2012 update (1), he found a lower rate of Grade 1 rectal and urinary toxicity with QOD compared to QD, but no significant difference in Grade 2–3. In addition, his total number of patients is fairly low at 67, making it difficult to draw any definitive conclusions. Therefore, contrary to popular belief, we feel it is not established that QOD treatments yield less rectal or urinary toxicity. Particularly with treatment delivered daily to 35 Gy, we shown very low rates of Grade 2–3 toxicity, with high efficacy. Our data, with many more patients, suggest that dose, rather than the schedule, is the most important predictor of late rectal toxicity.

Prostate SBRT has the added advantages of patient convenience and decreased healthcare cost. Only 5 treatment visits are required, in contrast to 40–45 for conventionally fractionated radiation. Furthermore, the current Medicare rate is substantially lower for prostate SBRT, which costs $21,000 in contrast to $32,000 for IMRT and $53,000 for protons.

## Conclusion

Cyberknife SBRT produces excellent long-term biochemical control rates with acceptably low rates of long-term toxicity, similar to other radiotherapy modalities. Our results suggest that 35 Gy is as effective as 36.25 Gy for low- and low-intermediate-risk patients, and has significantly less late Grade 2–3 urinary toxicity. Further follow-up extending beyond 10 years will be obtained to determine the durability of response and incidence of late toxicity. Both prospective and randomized studies are indicated to confirm these encouraging results.

## Conflict of Interest Statement

The authors declare that the research was conducted in the absence of any commercial or financial relationships that could be construed as a potential conflict of interest.
